# Numerous but Rare: An Exploration of Magic Squares

**DOI:** 10.1371/journal.pone.0125062

**Published:** 2015-05-14

**Authors:** Akimasa Kitajima, Macoto Kikuchi

**Affiliations:** 1 Research and Legislative Reference Bureau, National Diet Library, Chiyoda-ku, Tokyo, Japan; 2 Department of Physics, Graduate School of Science, Osaka university, Toyonaka, Osaka, Japan; 3 Large-Scale Computational Science Division, Cybermedia center, Osaka University, Toyonaka, Osaka, Japan; Max Planck Institute for the Physics of Complex Systems, GERMANY

## Abstract

How rare are magic squares? So far, the exact number of magic squares of order *n* is only known for *n* ≤ 5. For larger squares, we need statistical approaches for estimating the number. For this purpose, we formulated the problem as a combinatorial optimization problem and applied the Multicanonical Monte Carlo method (MMC), which has been developed in the field of computational statistical physics. Among all the possible arrangements of the numbers 1; 2, …, *n* ^2^ in an *n* × *n* square, the probability of finding a magic square decreases faster than the exponential of *n*. We estimated the number of magic squares for *n* ≤ 30. The number of magic squares for *n* = 30 was estimated to be 6.56(29) × 10^2056^ and the corresponding probability is as small as 10−^212^. Thus the MMC is effective for counting very rare configurations.

## Introduction

Making magic squares is a popular form of mathematical recreation. It is also used in classrooms as an elementary mathematical exercise. The classic (or ordinary) magic square of order *n* is defined as follows: Placing the numbers 1, 2, ⋯*n* ^2^ in a square array using each number once, if all the sums of the numbers in each row, column and diagonal give the same value, $M_{n}=\frac{1}{2}n(n^{2}+1)$, the array makes a magic square. *M*_*n*_ is called the magic number. Besides the classic magic squares, there are many variations, and some rigorous results have been found for them. But not much is known about classic magic squares [1]. In this paper, we focus on classic magic squares.

There are some algorithms for making magic squares of any size. They, however, provide some special classes of magic squares, which gives rise to the question: Among all the possible arrangements of numbers in a square of a given size, how many of them form magic squares? Putting the question in another form: Is there any chance of making a magic square by putting numbers randomly in a square? It may be surprising to know that the exact number of possible magic squares is so far only known up to order 5. There is currently no hope of exact enumeration for a larger system. In this paper, we apply a Monte Carlo method to this problem, and estimate the number of the magic squares of each size up to order 30.

To state the problem explicitly, we consider classic magic squares order *n*. Let *N*_*n*_ denote the total number of magic squares. Since possible configurations increase as *n* ^2^!, counting the magic squares rapidly becomes more difficult. Currently, only the following three values of *N*_*n*_ are known exactly: *N*_3_ = 1, *N*_4_ = 880, and *N*_5_ = 275,305,224 [1], where the eight equivalent magic squares that can be transformed into each other by rotation and reflection are counted as one.

For larger squares, we need to employ statistical approaches for estimating the number of magic squares. There have been two studies in this direction. Pinn and Wieczerkowski applied the exchange Monte Carlo method (EMC) [2] to this problem [3] and estimated *N*_6_ and *N*_7_. Their results are *N*_6_ = 1.7745(16) × 10^19^ and *N*_7_ = 3.760(52) × 10^34^, where the digits in parentheses indicate the statistical error of the lowest digits. Trump proposed a more efficient method, called Monte Carlo Backtracking, and estimated *N*_*n*_ for *n* ≤ 20. [4].

EMC belongs to a family of extended ensemble Monte Carlo methods [5]. Extended ensemble Monte Carlo methods were initially developed in the field of statistical physics, and have found a wide field of applications beyond their original scope. They are especially suitable for estimating the probability of occurrence of very rare events. The work by Pinn and Wieczerkowski is one of the earliest applications of the extended ensemble Monte Carlo methods outside the field of physics. In this paper, we use the Multicanonical Monte Carlo method (MMC) [6], which also belongs to a family of extended ensemble methods. There have also been some studies that used EMC for counting solutions for mathematical puzzles such as the N-Queen problem [7][8]. But the MMC has not been used for problems of this type. Compared to the EMC, which requires a trick for counting the number of configurations that satisfy some specific conditions, the MMC provides the estimates of the number straightforwardly. We thus consider the MMC to be more suitable for problems of this sort than the EMC.

## Methods

Let us consider magic squares of order *n*. In order to apply the MMC, we define the *energy* *E*(*C*) of a configuration of numbers *C* as follows:

$$\begin{array}{c} E\left(C\right)=\sum_{\text{columns}\;i}|M_{n}-S_{i}|+\sum_{\text{rows}\;j}|M_{n}-S_{j}|+\sum_{\text{diagonals}\;k}|M_{n}-S_{k}|, \end{array}$$ (1)

where *S*_*i*_, *S*_*j*_, and *S*_*k*_ are the sums of the numbers for the *i*th column, that for the *j*th row, and that for the *k*th diagonal. *E*(*C*) is zero if and only if *C* is a magic square, and it takes a positive value otherwise. Thus, the lowest energy (*E* = 0) configurations provide magic squares. In other words, finding problem of magic squares are formulated as a combinatorial optimization problem. The number of optimal configurations are very large in this case, and we estimate the number using the MMC.

The MMC was proposed by Berg and Neuhaus in the field of statistical physics to overcome slow convergence of Metropolis-type Markov chain Monte Carlo methods when applied to the sampling of low temperature states of complex systems [6]. In contrast to the Metropolis method which provides the canonical ensemble, namely the ensemble of configurations at the thermal equilibrium of a given temperature *T* as the steady-state distribution of the Markov process, the MMC aims to give a flat energy distribution over the entire energy range. This flatness enables us to estimate the number of configurations of any energy. For that purpose, a predetermined weight function *W*(*E*) is used in the MMC instead of the canonical weight *e* ^−*E*/*T*^ used in the Metropolis method. *W*(*E*) is prepared so that the energy histogram *H*(*E*) obtained by Monte Carlo sampling is sufficiently flat. Since *H*(*E*) is proportional to the product of *W*(*E*) and the number of states *g*(*E*) having energy *E*, we can then estimate relative values of *g*(*E*) from *W*(*E*) and *H*(*E*) as

$$\begin{array}{c} g\left(E\right)\propto \frac{H(E)}{W(E)}. \end{array}$$ (2)

The appearance probability of energy *E* = *ɛ* in randomly arranged configurations of numbers from 1 to *n* ^2^ is estimated by

$$\begin{array}{c} P\left(E=\varepsilon \right)=\frac{g(\varepsilon )}{\sum_{E}g\left(E\right)}, \end{array}$$ (3)

where the summation in the denominator is taken over all the possible energies. Thus the appearance probability of magic square *P*_*n*_ is given by *P*_*n*_ = *P*(*E* = 0). Since the total number of configurations is *n* ^2^!, we can also estimate the total number of magic squares *N*_*n*_ by *P*_*n*_ × *n* ^2^!/8. It should be noted that, in principle, MMC gives statistically unbiased estimates for the number of magic squares.

To determine *W*(*E*), we carried out preliminary runs using the Wang–Landau method [9], in which *W*(*E*) is updated at each Monte Carlo trial until it finally gives a sufficiently flat histogram *H*(*E*). We then fixed *W*(*E*) and carried out long measurement runs using the entropic sampling method [10], which is equivalent to MMC in the present study because we assigned a single value of energy to each bin of the histogram. We made independent measurement runs many times for each *n*, and took averages of *N*_*n*_ and $N_{n}^{2}$ over them. The statistical error of *N*_*n*_ was then estimated as three times the standard error:

$$\begin{array}{c} 3\sqrt{\frac{\left\langle N_{n}^{2}\right\rangle -{\left\langle N_{n}\right\rangle }^{2}}{t-1}}, \end{array}$$ (4)

where ⟨⋅⟩ is the mean value of ⋅ and *t* is the number of the measurement runs.

Only the sequential transposition of adjacent numbers, 1 with 2, then 2 with 3, ⋯, *n* ^2^−1 with *n* ^2^ were used as an elementary process of the Monte Carlo trial, following Ref. [3]. By this method, we can avoid a large energy difference between successive configurations in Markov chains, which causes inefficiencies in Monte Carlo methods. We employed Mersenne-Twister as the pseudo-random-number generator [11].

The number of measurement runs and the length of each run are different for each *n*. For the largest system with *n* = 30, for example, we made 40 independent measurement runs of 1.1 × 10^12^ Monte Carlo trials each. Flatness of the histogram was confirmed by a long independent run that was four times longer than the measurement run. The number of configurations in each bin of the histogram falls within the range from 0.93 to 1.01 of the mean value, which we decided as sufficiently flat.

## Results

**Fig 1:**
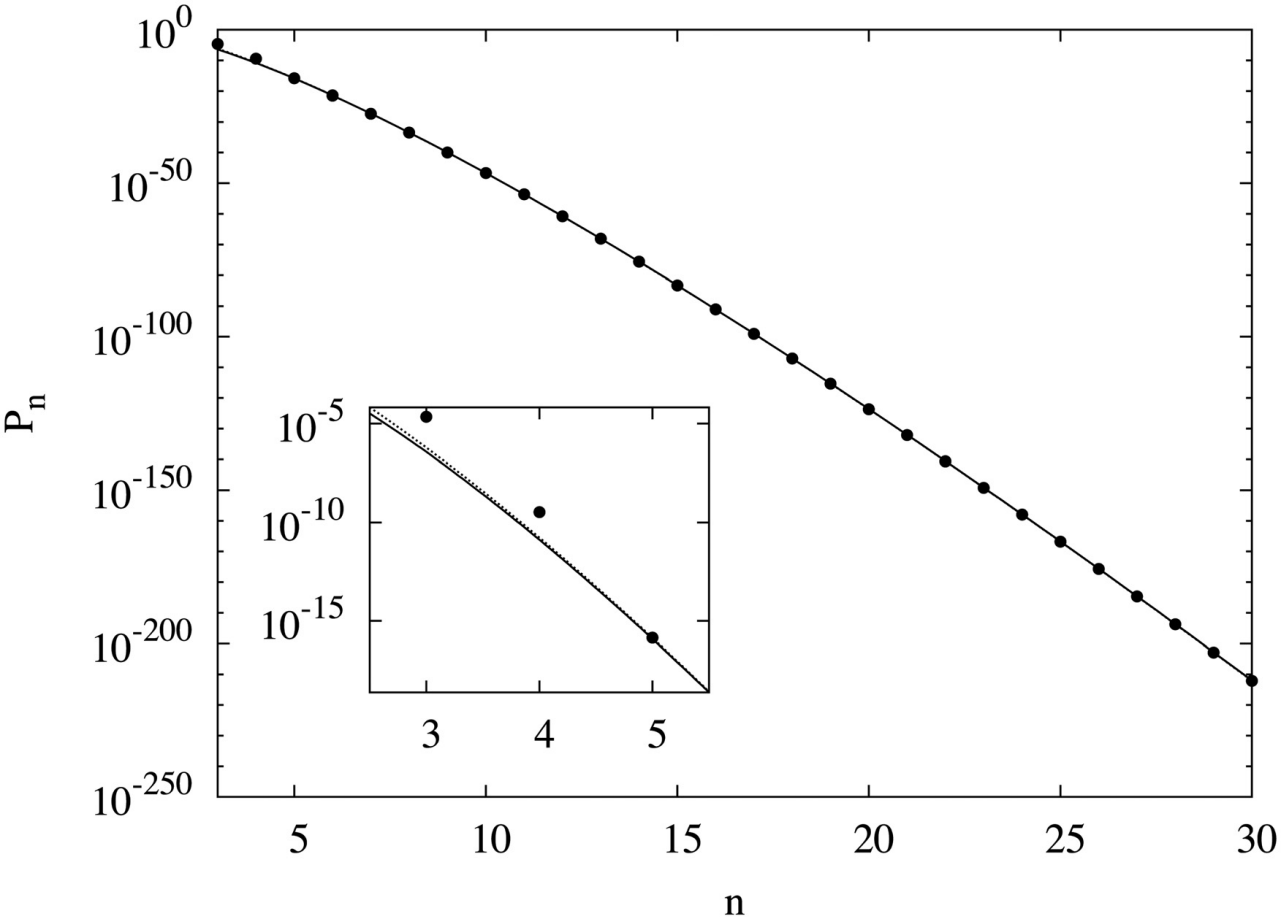
Semi-log plot of the appearance probability *P*_*n*_ of magic squares (•). *P*_*n*_ decreases faster than exponentially with the size *n*. Two fitted functions are also shown: exp((*An* + *B*)ln(*n*) + *Cn* + *D*) (solid line) and exp((*En* + *F*)ln(*n* + *G*) + *H*) (dotted line) with *A* = −4.99 and *E* = −4.88. We used *P*_*n*_ of *n* ≥ 10 for the fitting. Enlarged plot for *n* < 6 is shown in the inset, in which difference of two functions are visible.

Fig 1 shows a semi-log plot of the *n* dependence of *P*_*n*_. Our estimates of *P*_*n*_ and *N*_*n*_ up to *n* = 30 are listed in Table 1. Exact values for of *N*_*n*_ for *n* = 3,4 and 5 and the previous estimates of *N*_*n*_ by Trump for 6 ≤ *n* ≤ 20 are also shown for comparison. We obtained *N*_30_ = 6.56(29) × 10^2056^. In contrast to this extremely large value, its appearance probability, *P*_30_, is smaller than 10^−212^. Thus the magic squares are numerous but rare.

**Table 1:** Estimated number and qppearance probability of magic squares.

*n*	*P*_*n*_	*N*_*n*_	Trump’s estimates (* exact)
3	2.204(35) × 10^−5^	0.999(16)	1 *
4	3.3645(15) × 10^−10^	8.7995(39) × 10^2^	880 *
5	1.42011(88) × 10^−16^	2.7534(17) × 10^8^	275305224 *
6	3.8182(15) × 10^−22^	1.77543(73) × 10^19^	1.775399(42) × 10^19^
7	4.9955(92) × 10^−28^	3.7983(70) × 10^34^	3.79809(50) × 10^34^
8	3.2931(91) × 10^−34^	5.223(14) × 10^54^	5.2225(18) × 10^54^
9	1.0831(30) × 10^−40^	7.848(22) × 10^79^	7.8448(38) × 10^79^
10	2.069(14) × 10^−47^	2.414(17) × 10^110^	2.4149(12) × 10^110^
11	2.312(12) × 10^−54^	2.339(12) × 10^146^	2.3358(14) × 10^146^
12	1.645(10) × 10^−61^	1.1417(72) × 10^188^	1.1424(10) × 10^188^
13	7.564(61) × 10^−69^	4.036(32) × 10^235^	4.0333(54) × 10^235^
14	2.376(27) × 10^−76^	1.509(17) × 10^289^	1.5057(24) × 10^289^
15	5.082(66) × 10^−84^	8.00(10) × 10^348^	8.052(22) × 10^348^
16	7.933(98) × 10^−92^	8.50(11) × 10^414^	8.509(27) × 10^414^
17	8.898(61) × 10^−100^	2.313(16) × 10^487^	2.314(9) × 10^487^
18	7.500(66) × 10^−108^	2.146(19) × 10^566^	2.047(8) × 10^566^
19	4.657(86) × 10^−116^	8.37(15) × 10^651^	8.110(35) × 10^651^
20	2.216(50) × 10^−124^	1.773(40) × 10^744^	1.810(8) × 10^744^
21	8.34(24) × 10^−133^	2.589(73) × 10^843^	
22	2.503(73) × 10^−141^	3.189(93) × 10^949^	
23	5.88(21) × 10^−150^	3.92(14) × 10^1062^	
24	1.099(38) × 10^−158^	5.85(20) × 10^1182^	
25	1.640(44) × 10^−167^	1.258(34) × 10^1310^	
26	2.098(43) × 10^−176^	4.94(10) × 10^1444^	
27	2.150(62) × 10^−185^	3.86(11) × 10^1586^	
28	1.804(74) × 10^−194^	7.18(29) × 10^1735^	
29	1.276(61) × 10^−203^	3.77(18) × 10^1892^	
30	7.78(35) × 10^−213^	6.56(29) × 10^2056^	

Numbers in the parentheses indicate the statistical errors (3 times the standard error) in the last digits.

The largest size of *n* = 30 is much larger than that which has been calculated previously. The estimates of *N*_3_, *N*_4_, and *N*_5_ agree with the exact values within the statistical error, and the estimates up to *n* = 17 are consistent with Trump’s values within the statistical error. However, there are appreciable discrepancies between the present results and those of Trump for *n* = 18 and 19; our values are larger for these sizes. In fact, Trump himself pointed out that the true values for *N*_18_ and *N*_19_ might be smaller than his estimates based on his own extrapolation formula. We thus think that our estimates are reliable. He also gave estimates of *N*_*n*_ for *n* > 20 obtained by the abovementioned extrapolation formula. Compared to the present estimates, his extrapolation results have two-digits accuracy up to *n* = 30.

As seen in Fig 1, the appearance probability of magic squares *P*_*n*_ decreases rapidly with *n*. In other words, magic squares become rarer rapidly as *n* increases. This raises the question: how fast does their appearance probability decrease? It clearly decreases faster than the exponential function exp(−*an*) with constant *a*. On the other hand, since the number of possible configurations is *n* ^2^!, *P*_*n*_ should decrease slower than exp(−*n* ^2^log*n*). Considering these facts, we first tried to fit log*P*_*n*_ for *n* ≥ 10 by the second-order polynomial. But the reduced *χ* ^2^ was larger than 6000 and thus the fitting function was inappropriate. Next we tried functions including log*n*. The fitting function (*An* + *B*)log*n* + *Cn* + *D* with the constants *A*, *B*, *C* and *D* gave *A* = −4.99 ± 0.07 with the reduced *χ* ^2^ = 2.55 and the fitting function (*En* + *F*)log(*n* + *G*) + *H* with the constants *E*, *F*, *G* and *H* gave *E* = −4.880 ± 0.008 with the reduced *χ* ^2^ = 2.42. Fitted curves using these two functions are shown in Fig 1. The two curves are virtually indistinguishable on this scale except for very small values of *n*. Other functions we tried gave larger values of reduced *χ* ^2^. The reduced *χ* ^2^ for both functions, however, are still large, and the fittings are not fully satisfactory. We consider the reason is that *n* = 10 is not yet at the asymptotic region. In fact, when we tried to fit the same functions to data only for larger sizes, *n* ≥ 20, we obtained the reduced *χ* ^2^ = 1.30 and 1.37, respectively. Although the errors of the parameters are large, *A* = −4.6 ± 0.5 and *E* = −4.88 ± 0.06, reduced *χ* ^2^ for both functions are rather satisfactory.

**Fig 2:**
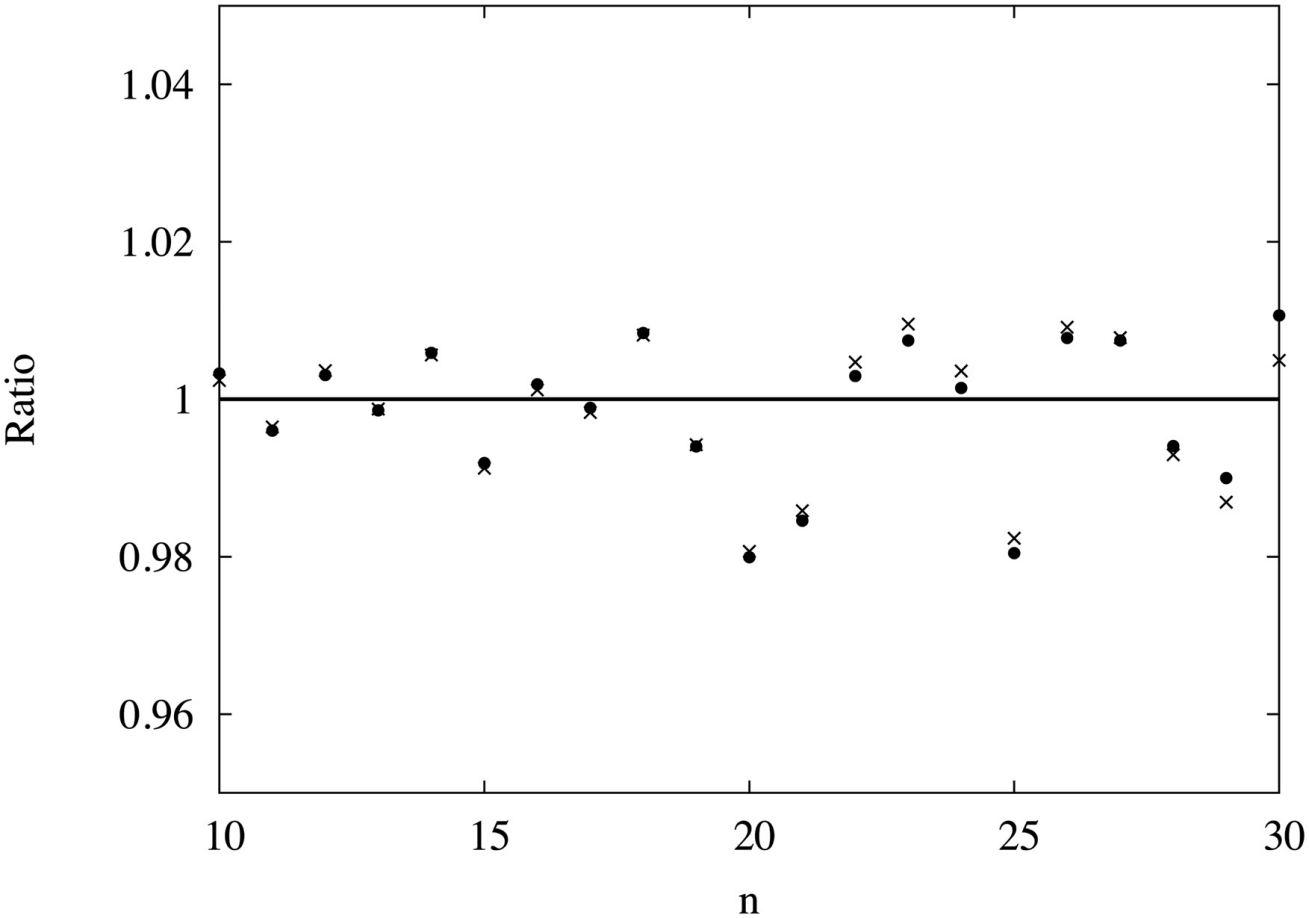
The ratio of the appearance probability *P*_*n*_ to two fitted functions. *P*_*n*_/exp{(*An* + *B*)ln(*n*) + *Cn* + *D*}} (•) and *P*_*n*_/exp{(*En* + *F*)ln(*n* + *G*) + *H*} (×) with *A* = −4.99 and *E* = −4.88. Both functions seem to express *P*_*n*_ equally well.

Fig 2 shows the ratio *P*_*n*_/exp{(*An* + *B*)log*n* + *Cn* + *D*} and *P*_*n*_/exp{(*En* + *F*)log(*n* + *G*) + *H*}. Both functions seem to express *P*_*n*_ equally well. In any case, since the slope of log*P*_*n*_ varies slowly, it is difficult to determine the appropriate functional form from the present results. We need *P*_*n*_ for much larger systems to provide a solid conclusion. Even so, we may conjecture that the log*P*_*n*_ decreases asymptotically as *an* log *n* with *a* ≃ 5.

In this paper, we applied the Multicanonical Monte Carlo method to a combinatorial optimization problem by defining appropriate an energy function *E*(*C*). The MMC directly gives the number of the optimal configurations from the histogram of the lowest energy configurations. The present work demonstrates that the MMC is a powerful tool for counting rare configurations of combinatorial problems. We can estimate the appearance probabilities of the optimal configurations as small as 10^−212^.
